# Myelination of Axons Corresponds with Faster Transmission Speed in the Prefrontal Cortex of Developing Male Rats

**DOI:** 10.1523/ENEURO.0203-18.2018

**Published:** 2018-09-13

**Authors:** Sean McDougall, Wanette Vargas Riad, Andrea Silva-Gotay, Elizabeth R. Tavares, Divya Harpalani, Geng-Lin Li, Heather N. Richardson

**Affiliations:** 1 Department of Psychological and Brain Sciences; 2 Neuroscience and Behavior Graduate Program; 3 Biology Department, University of Massachusetts, Amherst, MA 01003

**Keywords:** anterior cingulate, conduction velocity, forceps minor, g-ratio, myelin whole-cell patch clamp

## Abstract

Myelination of prefrontal circuits during adolescence is thought to lead to enhanced cognitive processing and improved behavioral control. However, while standard neuroimaging techniques commonly used in human and animal studies can measure large white matter bundles and residual conduction speed, they cannot directly measure myelination of individual axons or how fast electrical signals travel along these axons. Here we focused on a specific population of prefrontal axons to directly measure conduction velocity and myelin microstructure in developing male rats. An *in vitro* electrophysiological approach enabled us to isolate monosynaptic projections from the anterior branches of the corpus callosum (corpus callosum-forceps minor, CC_FM_) to the anterior cingulate subregion of the medial prefrontal cortex (Cg1) and to measure the speed and direction of action potentials propagating along these axons. We found that a large number of axons projecting from the CC_FM_ to neurons in Layer V of Cg1 are ensheathed with myelin between pre-adolescence [postnatal day (PD)15] and mid-adolescence (PD43). This robust increase in axonal myelination is accompanied by a near doubling of transmission speed. As there was no age difference in the diameter of these axons, myelin is likely the driving force behind faster transmission of electrical signals in older animals. These developmental changes in axonal microstructure and physiology may extend to other axonal populations as well, and could underlie some of the improvements in cognitive processing between childhood and adolescence.

## Visual Abstract

**Figure:**
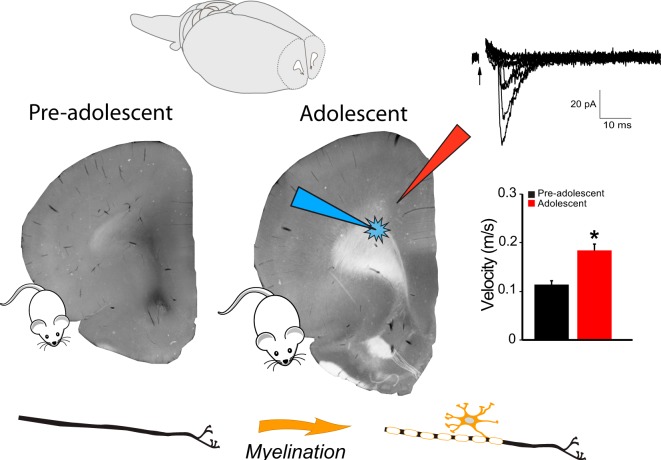

## Significance Statement

Neural processing improves during childhood and adolescent development, but the specific factors contributing to these developmental changes are largely unknown. The present study shows that between two and six weeks of age in male rats, axons in the prefrontal cortex undergo microstructural and electrophysiological changes that speed up neural transmission. These axonal changes could contribute to some of the developmental improvements in behavioral control and cognitive abilities dependent on the prefrontal cortex.

## Introduction

Cognitive abilities and behavioral control improve significantly during childhood and adolescent development (Casey et al., 2000; Lenroot and Giedd, 2006). To understand how these functions improve, we must first identify the factors underlying enhanced neural processing in the medial prefrontal cortex (mPFC) of the maturing brain. This is a brain region that integrates information from multiple sources to process complex functions including affective perception of pain (Fuchs et al., 2014), modulation of stress responses (Law et al., 2009), behavioral control (Takenouchi et al., 1999), attention (Rushworth et al., 2003; Kaping et al., 2011), and working memory (Seamans et al., 1995). Physical changes to axonal pathways projecting into the mPFC could contribute to improved prefrontal functions in adulthood (Chugani et al., 1987; Paus et al., 1999; Sowell et al., 1999). Here, we sought to determine when and how prefrontal axons change in developing male rats.

Magnetic resonance imaging studies in humans have shown that white matter, which contains a large number of myelinated fiber tracts, increases in volume in the frontal cortex between childhood and adulthood (Bartzokis et al., 2001; Perrin et al., 2008). These macrostructural changes correspond with increased cognitive abilities (O’Muircheartaigh et al., 2014; Deoni et al., 2016). Myelination of axons during development may enhance neural processing because this lipid-rich coating provides insulation that facilitates the propagation of action potentials along the axons via saltatory conduction (Fuster, 2002; Giedd, 2004). Indeed, increases in indices of myelination like fractional anisotropy (FA) and myelin volume fraction during development have been associated with higher processing speed, as measured by apparent (“residual”) conduction speed and inspection times (Dubois et al., 2008; Chevalier et al., 2015).

It should be noted, however, that FA is a measurement sensitive to many tissue properties aside from myelination, including axonal orientation and density (Jones et al., 2013). Myelination of axons within this pathway could potentially lead to faster neurotransmission, but this is not known because electroencephalograms are only able to assess the amount of time it takes for a sensory stimulus to elicit a neural response. Thus, faster processing of the stimulus could be due either to increased conduction velocity along individual axons or to other factors changing in the multisynaptic pathway during development. Few studies have measured how fast electrical signals travel along individual axons to directly test how neurotransmission changes when axons are myelinated in the developing prefrontal cortex.

In addition to overall increases in transmission speed, myelination of axons during development could also improve synchronicity in the timing of signals coming into the prefrontal cortex from other brain regions. Isochronicity describes electrical signals arriving at the same time to a target, despite differences in the length of axonal projections to that target (Sugihara et al., 1993; Pelletier and Paré, 2002; Salami et al., 2003). Isochronicity has been observed in neural input coming from different thalamic regions to the somatosensory cortex and this is thought to be due to differential myelination of these two axonal populations (Salami et al., 2003; Kimura and Itami, 2009). Notably, myelin ensheathment is complex and can vary in length and thickness along the same axon depending on where it resides within the brain (Tomassy et al., 2014). It is therefore important to examine myelin microstructure along different portions of axons that reside in white versus gray matter regions of the brain.

The present study investigated how prefrontal axons change physically and functionally during development. To accomplish this goal, we focused on an axonal population that extends from the anterior branches of the corpus callosum (corpus callosum-forceps minor, CC_FM_) into the dorsal mPFC (anterior cingulate cortex, Cg1). The Cg1 is not fully developed by adolescence (Benes, 1989; Cunningham et al., 2002, 2008) and has been shown to be critical for attention (Koike et al., 2016), spatial memory (Wartman et al., 2014), and decision-making (Kennerley et al., 2006). We also found myelinated axons in this region are sensitive to alcohol (Vargas et al., 2014). Thus, the location of the Cg1 respective to the CC_FM_ provides a unique experimental preparation to isolate, measure, and analyze different physical and electrophysiological properties of these axons in developing animals. We used a two-pronged approach to study these axons in pre-adolescent and adolescent male rats. Neurotransmission speed of individual axons was assessed by slice electrophysiology and myelin ensheathment was assessed using microstructural histology. This strategy enabled us to detect age-dependent changes in myelin and faster neural processing within individual axons that could contribute to improved neural processing in a brain region critically important for executive functions and behavioral control.

## Materials and Methods

### Animals

Male Wistar rats were ordered from Charles River (pre-adolescent rats were shipped with nursing moms). Separate animals were used for the electrophysiological and myelin microstructure histological experiments. For the electrophysiological experiments, “pre-adolescent” animals were postnatal day (PD)8–PD15 and “adolescent” animals were PD40–PD58. In these experiments, 21 total cells were recorded from five pre-adolescent rats and 13 total cells were recorded from four adolescent rats. For the myelin microstructure histological experiments, brains were processed from four pre-adolescent (PD15) and four adolescent (PD43) animals. All animals were kept on a 12/12 h light/dark cycle (lights on at 8 A.M.), with food and water available ad libitum. Weaning of pups does not occur until PD21; thus, all pre-adolescent animals were housed with nursing moms and three to four other pups, whereas adolescent animals were housed two to three per cage in this study. All procedures were performed according to the National Institutes of Health Guide for the Care and Use of Laboratory Animals and approved by the Institutional Animal Care and Use Committee.

### Experiment 1: *in vitr*o slice electrophysiology

#### Preparation of brain tissue slices for electrophysiology

Following CO_2_ euthanasia, brains were rapidly removed and placed into ice-cold cutting solution, which contained 89.1 mM sucrose, 13.88 mM glucose, 87.27 mM NaCl, 2.48 mM KCl, 1.25 mM sodium phosphate monobasic monohydrate, 25 mM sodium bicarbonate, 7 mM MgCl_2_·6H_2_O, and 0.37 mM CaCl_2_. Coronal slices were cut at 300-µm thickness with a vibratome (Leica VT1200 S with Vibrocheck). The slices were then incubated in artificial CSF (aCSF) at 33°C for 45 min. aCSF contained 127 mM NaCl, 25 mM sodium bicarbonate, 25 mM glucose, 2.5 mM KCl, and 1.25 mM sodium phosphate monobasic monohydrate. The cutting solution and aCSF were bubbled with 95% O_2_ and 5% CO_2_ gas.

#### Stimulation and recordings for electrophysiology

**Figure 1.**
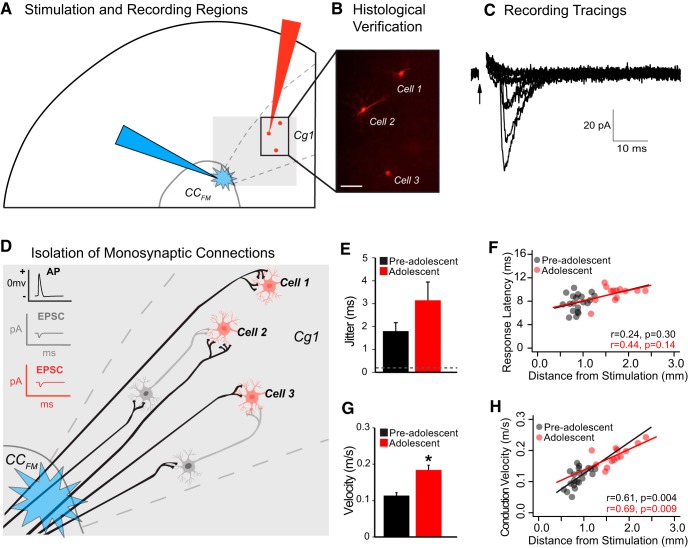
Developmental increase in transmission speed along axons extending from the anterior corpus callosum (CC_FM_) to the cingulate cortex (Cg1). ***A***, Schematic drawing of a coronal section illustrating placement of the stimulating electrode (blue) in the CC_FM_ and recording electrode (red) in the Cg1. ***B***, Histological verification of neurons recorded from the Cg1 (scale bar = 50 μm). ***C***, Sample recording showing a series of 10 superimposed traces taken from a single cell using a constant amplitude threshold stimulation. Responses did not differ in the maximum amplitude of their current, but were still all-or-none, seen by the frequent instances when stimulation failed to evoke any responses from the cell. ***D***, Schematic illustrating the minimum threshold stimulation design, which isolates axons sending monosynaptic connections to recorded cells in the cortex. In this image, axons in the CC_FM_ are stimulated (black) and neurons in the cortex (red) are patch clamped. These CC_FM_ axons synapse onto both the recorded cells (red) and onto non-recorded cells (gray). The gray cells illustrate intermediate cells that are postsynaptic to the CC_FM_ axons, but presynaptic to the patch clamped red cells. The low intensity stimulation paradigm is not strong enough to evoke an action potential in these postsynaptic gray cells. Therefore, only the direct (monosynaptic) connections determine the EPSCs detected in the red recorded cells. ***E***, Jitter was calculated to be above 0.2 ms in both pre-adolescent and adolescent groups; therefore, conduction was orthodromic, i.e., traveling down the axon toward the terminals. ***F***, There was no correlation between latency and transmission distance detected in either age group (pre-adolescent: *r* = 0.24, *p* = 0.30; adolescent: *r* = 0.44, *p* = 0.14). ***G***, There was a significant increase in the average conduction velocity between pre-adolescence and adolescence (*t*_(24.68)_ = 6.47, *p < 0.001). Note: conduction velocity calculation includes synaptic delay. ***H***, Velocity correlated with transmission distance in both age groups (pre-adolescent: *r* = 0.61, *p* = 0.004; adolescent: *r* = 0.69, *p* = 0.009). Electrophysiological data (obtained from 21 cells from five pre-adolescent rats and 13 cells from four adolescent rats) are expressed as mean ± SEM. Illustrations were created by modifying images purchased in the PPT Drawing Toolkits-BIOLOGY Bundle from Motifolio, Inc.

Slices were transferred to a recording chamber and continuously perfused with aCSF. During recording, neurons were located using an Olympus BX51W1 microscope with 4× and 60× objectives. Cells in the Cg1 were then patched under the whole-cell voltage-clamp mode (the amplifier was HEKA EPC10/2 USB with Patch master for data acquisition). Micropipettes had a bath resistance between 3 and 11 MΩ (pipette puller Narishige PC-10), with an internal solution containing 130 mM KGlu, 10 mM KCl, 10 mM HEPES, 1 mM EGTA, 3 mM MgATP, and 0.5 mM NaGTP combined with Alexa Fluor 594 fluorescent dye to visualize the patched cells (Fig. 1*B*). It is important to note that while Alexa Fluor 594 dye has been shown to alter AMPA receptor-mediated EPSCs at +40 mV, these effects were not evident at lower voltage (-60 mV, Maroteaux and Liu, 2016). It is therefore unlikely that this dye had a measurable effect on our results, as our cells were held at -80 mV (described below). A concentric bipolar stimulating electrode was used to excite fibers in the dorsal-medial region of the CC_FM_ (Analog Stimulus Isolator Model 2200 from A-M Systems). For all animals, the boundary between the CC_FM_ white matter and the beginning of the gray matter (Cg1) could easily be discerned under 60× magnification because fiber tracts were visible in CC_FM_, whereas no fiber tracts were visible in cortical tissue. The stimulation electrode was placed in the CC_FM_ and it remained there through the duration of the experiment, whereas the recording electrode was moved from one cortical neuron to another within the Cg1. EPSCs were recorded in the patched cells within the cortical gray matter under voltage clamp at -80 mV. We held the cells at this voltage to increase EPSC amplitudes so that EPSCs with small amplitudes could be detected reliably. Initially, a stimulation ramp protocol was run to determine how the EPSC profile changed with increasing stimulation voltage, and then the cell was repeatedly stimulated with a constant intensity to establish a stable response.

A low intensity stimulation paradigm (10–30 μA, 0.2 ms) was used to generate a minimal amplitude EPSC. This strategy was used for two reasons. First, in order for a cell to respond to such a low stimulation, the CC_FM_ axon stimulated must have a monosynaptic connection to the cell. This assumes that if there were a cell intermediate to the stimulated axon and the recorded cell, the low intensity stimulation of the axon would generate an EPSC in this intermediate cell, but this would not be sufficient to elicit an action potential that would then stimulate an EPSC in the patched cell (illustrated in Fig. 1*D*). Second, the axon terminal must have multiple synapse sites on the postsynaptic cell to generate a detectable change in current, i.e., the sum of the EPSCs delivered must be detectable above noise. This strategy therefore isolated the single axon that has the strongest connection to the recorded cell. Threshold was determined by lowering the stimulation intensity to the minimum level at which EPSCs could still be evoked, accompanied by frequent failures. The EPSC onset was measured as the time point where the slope of the current changed from baseline following the EPSC artifact.

#### Electrophysiological measurements and analyses

Images of the patched cells were taken under 4× magnification to determine the distance between the stimulation site and recorded cell (transmission distance). Two different methods were used to determine the response latency. The first method used the time difference between the start of the stimulation artifact (Fig. 1*C*, arrow) and the detection of the post-artifact current change that fell below the baseline current of the cell. The second method determined the latency between the start of the artifact and the peak of the resulting EPSC. This method was only used to calculate the response latency jitter for each cell, as the EPSC peak does not necessarily reflect the most accurate measure of EPSC onset. Jitter is the variability in response latency observed throughout various stimulations. A response latency jitter value higher than 0.2 ms indicates that the response is synaptically driven, or orthodromic (Pelletier and Paré, 2002), as neurotransmission time can vary between trials. Jitter for each series of stimulations was calculated as the standard deviation of the response latencies using ≥10 individual traces.

### Experiment 2: histology

#### Processing of brain tissue for analysis of myelin

**Figure 2.**
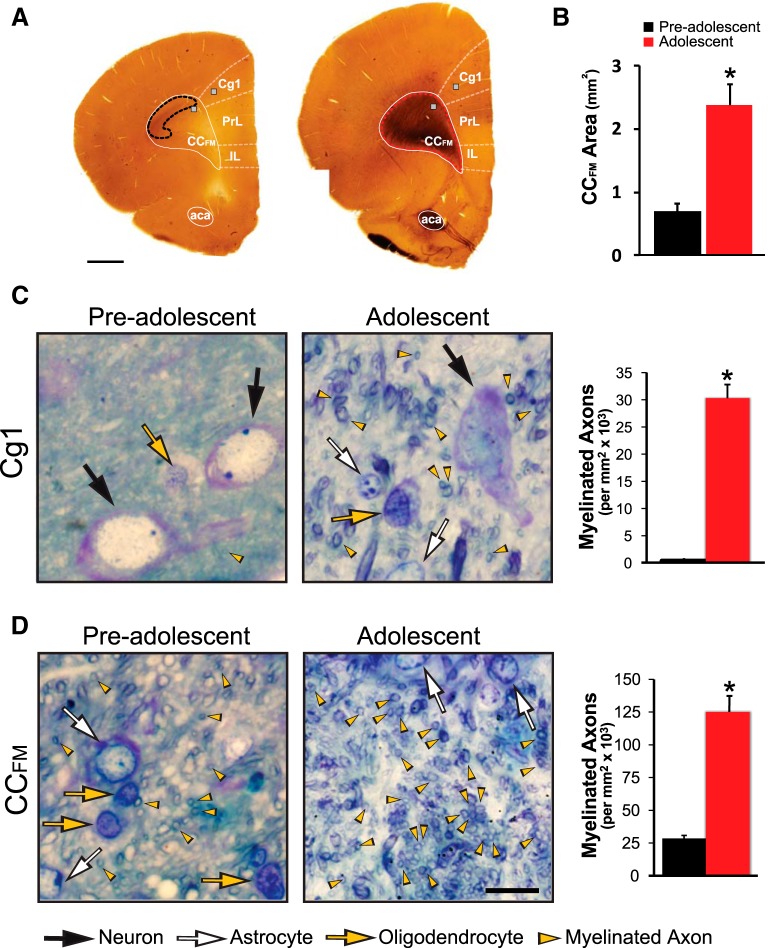
Developmental changes in macro- and microstructural measurements of myelin in the prefrontal cortex of male rats. ***A***, Embedded coronal brain tissue slices of a pre-adolescent rat (PD15, left) and an adolescent rat (PD43, right; scale bar = 1 mm). The myelinated portion of the CC_FM_ is outlined with a black dotted line in the pre-adolescent tissue slice and outlined with a red dotted line in the adolescent tissue slice to illustrate how myelinated cross-sectional area was analyzed. Small squares on the embedded slices indicate the location of the images shown in ***C***, ***D***. ***B***, There was a significant increase in the myelinated cross-sectional area of CC_FM_ in adolescent rats compared to pre-adolescent rats (*t*_(3.95)_ = 5.65, *p = 0.005). ***C***, ***D***, Representative images of myelin development over adolescence in Cg1 and CC_FM_ regions (scale bar = 20 μm). Neurons (black arrows), astrocytes (white arrows), and oligodendrocytes (orange arrows) were identified and distinguished using an algorithm created by García-Cabezas et al. (2016) that is based on cytological features. Examples of myelinated axons that crossed through the plane of section in the 1.5-μm semi-thin sections are labeled with small orange arrowheads. The number of myelinated axons and area-based g-ratio were determined from cross-sectioned axons such as these. Myelinated axons that were parallel to the plane of the semi-thin sections were not included in the analysis (not labeled in this figure). There was a significant developmental increase in the number of myelinated axons in Layer V of the Cg1 (***C***, *t*_(3.00)_ = 14.17, *p = 0.0008) and CC_FM_ (***D***, *t*_(3.29)_ = 8.88, *p = 0.002). Data expressed as mean ± SEM. PrL, prelimbic cortex; IL, infralimbic cortex; aca, anterior commissure.

Pre-adolescent (PD15) and adolescent (PD43) rats were perfused with 1% paraformaldehyde/1.25% glutaraldehyde in 0.12 M phosphate buffer, pH 7.4 with 0.2 mM CaCl_2_ added. After 4 d postfixation, a vibratome was used to cut 150-μm coronal slices containing the prefrontal cortex (3.20–1.85 mm from bregma; Paxinos and Watson, 1998). These slices were rinsed in 0.12 M phosphate buffer with 0.2 mM CaCl_2_ and 8% dextrose and postfixed in 2% osmium tetroxide. Following en bloc staining with uranyl acetate, the slices were dehydrated and embedded in Polybed 812 between two sheets of Aclar film. The phospholipids and myelin sheaths were clearly visible as a dark brown color in the slices after the osmium postfixation (Fig. 2*A*). For each embedded slice, five digital images were obtained using a brightfield Leica microscope (2.5× objective) attached to a DP71 Olympus camera. These digital images were then combined together in a mosaic using MosaicJ from ImageJ (Thévenaz and Unser, 2007) to reconstruct the full prefrontal section for measurement of CC_FM_ cross-sectional area. Next, a 0.8 × 1.2 mm region containing the dorsomedial CC_FM_ and adjacent Cg1 was dissected from each embedded slice. Dissected tissue samples were mounted on epoxy blocks and 2.5-µm semi-thin coronal sections were collected using a Sorvall JB 4 microtome. Semi-thin sections were then mounted on subbed glass slides, stained with 0.1 M toluidine blue, dehydrated, and coverslipped for microscopic analysis (described below).

#### Microscopic analysis of embedded slices and semi-thin sections

The digital mosaics of the embedded slices were used for macrostructural analysis of prefrontal white matter. ImageJ software (Rasband, 1997) was used to trace the boundaries of the CC_FM_ and measure the cross-sectional area. The semi-thin sections were used for all other myelin measures. Digital images were collected from Layer V of Cg1 within the mPFC at 2.7 mm anterior to bregma using a brightfield Leica microscope (100× oil objective) attached to a DP71 Olympus camera (Fig. 2*C*). We focused on Layer V because it is the main output layer of the mPFC (Goodfellow et al., 2009; van Aerde and Feldmeyer, 2015). Layer V was identified by its characteristic large pyramidal neurons with apical dendrites that extend through Layers II/III out to Layer I of the cortex (Kiernan and Hudson, 1991; Swenson, 2006; Wang et al., 2006; Fénelon et al., 2011; Vostrikov and Uranova, 2011). Images were also obtained from the dorsal medial edge of the CC_FM_ (Fig. 2*D*).

**Figure 3.**
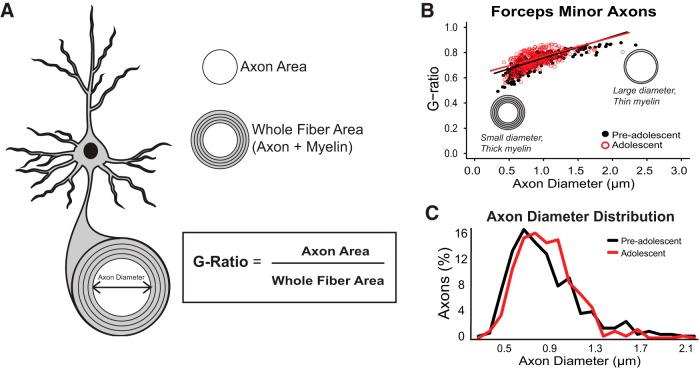
There were no developmental changes in axonal diameter or g-ratios of myelinated axons in the CC_FM_. ***A***, Schematic showing a cross-section of a myelinated axon. G-ratios were calculated by obtaining a measurement of the inner axon area and the whole fiber area. ***B***, Relative myelin thickness, as measured by g-ratio, did not change between pre-adolescence and adolescence (*n* = 4 per group, *t*_(5.43)_ = 0.78, *p* = 0.47). ***C***, A two sample Kolmogorov–Smirnov test showed that axon diameter distribution does not change from pre-adolescence to adolescence (*p* = 0.1708). Data are expressed as mean ± SEM. Illustrations were created by modifying images purchased in the PPT Drawing Toolkits-BIOLOGY Bundle from Motifolio, Inc.

The number of myelinated axons and the thickness of myelin sheaths relative to axonal size (g-ratio) were quantified (Figs. 2*C*,*D* and 3*B* ) using ImageJ software (Rasband, 1997) modified from Michailov et al. (2004). Axons were identified by matching morphologic characteristics, as described in previous studies (Peters et al., 2001; Chomiak and Hu, 2009; Liu et al., 2012). We quantified g-ratio in at least 100 randomly selected myelinated axons for each animal as an index of myelin thickness relative to fiber diameter. Only axons with their entire myelin sheaths visible were selected for measurements. Area-based g-ratio was used rather than standard diameter-based g-ratio because axons in the central nervous system are not perfectly circular in cross-sections (Almeida et al., 2011; Bakken and Stevens, 2012; Perge et al., 2012). The g-ratio was calculated by dividing the axon area by the area of the axon plus the myelin sheath combined (Fig. 3*A*).

### Statistical analyses

All statistical analyses were performed using R statistical software package (R Core Team, 2014). Student’s *t* tests were used to assess the difference in conduction velocity, jitter, forceps minor area, and myelinated axon number between the two age groups. To analyze g-ratio, the average g-ratio was calculated for each region in each rat by analyzing 100 axons for Cg1 and 100 axons for CC_FM_ in each animal. Groups were compared by Student’s *t* test using the average g-ratio of each rat. Pearson correlation analyses were used to test for relationships between latency and transmission distance, velocity and distance, and between g-ratio and axon diameter. A two-sample Kolmogorov–Smirnov test was used to compare axon diameter distributions between groups. Statistical significance was defined as *p* ≤ 0.05 using two-tailed tests.

## Results

### Conduction velocity increased from pre-adolescence to adolescence in CC_FM_ axons that synapse onto cells in Layer V of the Cg1

We evaluated how age impacted the speed of neurotransmission in CC_FM_ fibers with monosynaptic connections to cells in the Cg1. Conduction velocity (calculation includes synaptic delay) nearly doubled in these axons from pre-adolescence (PD8–PD15) to adolescence (PD40–PD58, 0.11 ± 0.01 vs 0.18 ± 0.01 m/s, respectively, *t*_(24.68)_ = 6.47, *p* = 0.000001; Fig. 1*G*). Importantly, jitter was above 0.2 ms for all cells (range: 0.26–7.91), indicating that the stimulation was orthodromic and traveled down the axon to the terminals in Layer V of Cg1 (Fig. 1*E*).

### There was a positive relationship between conduction velocity and transmission distance in both pre-adolescent and adolescent groups

To determine whether conduction velocity remains constant across axons or changes depending on how far away the postsynaptic cell is from the stimulation, we first analyzed the relationship between the time to EPSC onset (“response latency”) and the distance between the stimulation site and the recorded cell (“transmission distance”). These variables were not significantly correlated in either age group (pre-adolescents: *r* = 0.24, *p* = 0.30; adolescents: *r* = 0.44, *p* = 0.14; Fig. 1*F*). However, when we examined the relationship between conduction velocity and transmission distance, there was a significant, positive correlation in both pre-adolescents (*r* = 0.61, *p* = 0.004) and adolescents (*r* = 0.69, *p* = 0.0085). Thus, transmission speed was fastest in the axons that projected to cortical cells further away from the CC_FM_ (Fig. 1*H*).

### The number of myelinated axons increased from PD15 to PD43 in the Cg1 and CC_FM_

To address whether developmental increases in the speed of neurotransmission between the CC_FM_ and Cg1 corresponds to increased myelination in these regions, we first assessed if there were age-dependent changes in the cross-sectional area of the CC_FM_ (myelinated portion). The size of CC_FM_ was more than three times larger in adolescent compared to pre-adolescent rats (0.70 ± 0.13 vs 2.39 ± 0.32 mm^2^, respectively, *t*_(3.95)_ = 5.65, *p* = 0.005; Fig. 2*A*,*B*). The developmental increase in the size of the CC_FM_ size may be due in part to a near 5-fold increase in the density of myelinated axons in this structure (28.64 × 10^3^ ± 2.69 × 10^3^ axons per mm^2^ in pre-adolescent animals vs 125.15 × 10^3^ ± 12.26 × 10^3^ axons per mm^2^ in adolescent animals, *t*_(3.29)_ = 8.88, *p* = 0.002; Fig. 2*D*). The change in the number of myelinated axons was even more substantial in the Cg1, with a 90-fold increase in density from pre-adolescence to adolescence (0.33 × 10^3^ ± 0.07 × 10^3^ axons per mm^2^ vs 30.34 × 10^3^ ± 2.44 × 10^3^ axons per mm^2^, respectively, *t*_(3.00)_ = 14.17, *p* = 0.0008; Fig. 2*C*).

### No measurable changes in relative myelin thickness were detected in the CC_FM_ between PD15 and PD43

We next measured the average g-ratio of myelinated axons in the CC_FM_ of both age groups to determine if there were developmental increases in the relative thickness of myelin on these axons. G-ratios were not different between the two age groups, indicating that the relative thickness of myelin sheaths was not a factor contributing to the larger size of the CC_FM_ in older animals (0.73 ± 0.01 vs 0.74 ± 0.02, respectively, *t*_(5.43)_ = 0.78, *p* = 0.47; Fig. 3*B*). Based on the combined findings above (a developmental increase in the number of myelinated axons without an overall decrease in g-ratios), we assume that these CC_FM_ axons are undergoing *de novo* myelination and the process from initiation of wrapping to completion of a myelin segment occurs quite rapidly.

### The size (diameter) of myelinated axons in the CC_FM_ did not change with development

To address whether developmental increases in the speed of neurotransmission corresponds to increased axon diameter, we measured average myelinated axon diameter and assessed the distribution of diameters in CC_FM_ between PD15 and PD43. Average axon diameter did not change between the two age groups (0.89 ± 0.10 vs 0.89 ± 0.07 μm, respectively, *t*_(750.01)_ = -0.02, *p* = 0.98). Two-sample Kolmogorov–Smirnov test showed no significant changes between the distributions (*p* = 0.17). Thus, both the average diameter of axons and the range of axon diameters did not increase from pre-adolescence (range: 0.3–2.3 μm) to adolescence (range: 0.4–2.1 μm; Fig. 3*C*).

## Discussion

**Figure 4.**
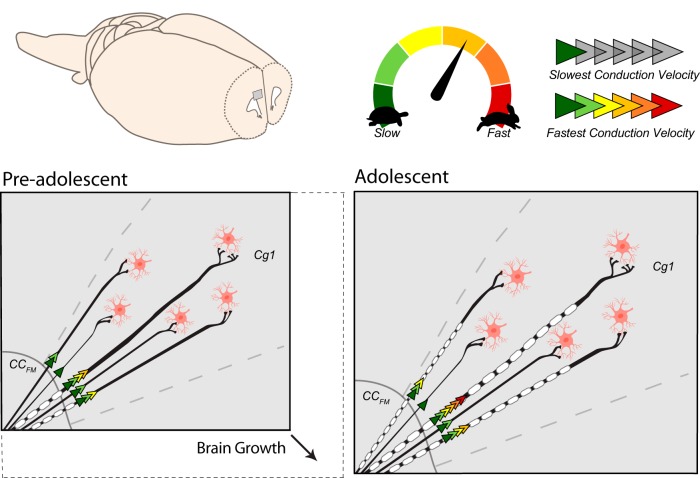
Theoretical model summarizing structural and functional changes in axons within the developing prefrontal cortex. Electrophysiological analyses revealed a significant developmental increase in neurotransmission speed in axons projecting from the CC_FM_ to Layer V in the Cg1 (speed is illustrated by the number and color of arrowheads). This increase in conduction velocity is accompanied by a developmental increase in the number of myelinated fibers and no change in axonal diameter, suggesting that myelination is a key factor driving faster neuronal processing. A positive relationship between conduction velocity and transmission distance in all animals suggests longer axonal projections may have a mechanism to ensure synchronized timing of incoming signals to Cg1 neurons. This phenomenon is observed even in the young age group, which has almost no myelin in these regions. Thus, other physical parameters such as axonal diameter, which varied widely among this axonal population, could serve to synchronize the timing of incoming information to postsynaptic cells residing in different locations in the Cg1. Illustrations were created by modifying images purchased in the PPT Drawing Toolkits-BIOLOGY Bundle from Motifolio, Inc.

The brain undergoes a series of maturational processes during childhood and adolescence that is thought to drive enhanced cognitive function in adulthood. To gain a better understanding of these developmental factors at the circuit level, we used histological and electrophysiological approaches to test for changes in axonal myelination and neurotransmission speed in the developing mPFC of male rats. Between two and six weeks of age, a large number of axons are being myelinated in this anterior region of the brain, and the addition of myelin segments along the axon appeared to move anterogradely from the corpus callosum out toward the axonal terminals in Layer V of the adjacent cortex (Fig. 4). These data complement previous studies investigating white matter changes in the developing brain of rodents (Kim and Juraska, 1997; Markham et al., 2007; Calabrese and Johnson, 2013; Downes and Mullins, 2014; Mengler et al., 2014; Willing and Juraska, 2015), and provide new evidence suggesting that increases in prefrontal white matter could be partially due to *de novo* myelination of axons rather than the thickening of myelin sheaths on previously-myelinated axons. Correspondent with myelination of prefrontal axons, was a significant increase in the speed at which electrical signals travel down these axons. By isolating monosynaptic projections, we gained insight into how electrical information travels from the anterior branches of the corpus callosum to the mPFC. The robust morphologic changes and improvements in transmission speed in individual prefrontal axons that were observed in the present study provide a means by which cognitive processing could improve between childhood and adolescence (Casey et al., 2000; Lenroot and Giedd, 2006).

Myelination of prefrontal axons appears to be a key factor underlying the developmental increases in neural processing. There was a significant increase in the number of myelinated axons in the CC_FM_ and Cg1 and the change in the size of the CC_FM_ in older animals is presumably due to *de novo* myelination of axons rather than thickening of myelin sheaths on previously-myelinated axons. Consistent with this interpretation, we did not find an age-dependent increase in the relative thickness of myelin (decrease in g-ratio). In older animals, electrical signals traveled twice as fast along axonal projections from the CC_FM_ to neurons in Layer V of the Cg1. Two different mechanisms could account for increased transmission speed: (1) larger diameter of the axons or (2) myelination of the axons (Hursh, 1939; Waxman, 1980; Hartline and Colman, 2007). As there was no age difference in the mean diameter or range of diameters in myelinated axons we can rule out increased diameter as a mechanism for increased axonal speed. Instead, myelination appears to be responsible for the developmental increase in axonal signal transmission speed.

To the best of our knowledge, this is the first demonstration of increased conduction velocity in this specific fiber population (CC_FM_ → Cg1), providing insight into one mechanism by which neural processing is enhanced during development. Perhaps similar increases in conduction velocity are occurring in axons within the posterior branches of the corpus callosum (splenium) and other regions that are being myelinated during this time (Kim and Juraska, 1997; Downes and Mullins, 2014; Mengler et al., 2014).

The data herein allow us to make inferences about how the myelination process takes place in the juvenile prefrontal cortex. Myelin ensheathment of prefrontal axons during this developmental period seems to move from the more lateral portion in white matter to the more medial portion in gray matter. In our sampled region, we observed that >28,000 axons/mm^2^ were myelinated in the CC_FM_ of PD15 animals, but only 334 axons/mm^2^ were myelinated in the Cg1 at this age. By PD43, the number of myelinated axons increased dramatically in the Cg1, reaching over 30,000 axons/mm^2^. Based on our electrophysiological evidence in monosynaptic axonal projections and macroscopic visualization of these axonal projections in embedded slices, we assume the microstructural myelin parameters obtained from the CC_FM_ versus the Cg1 serve as representative examples of white versus gray matter segments of same group of axons. Presumably, oligodendrocytes are adding myelin sheath segments along these prefrontal axons moving from the lateral portions in the white matter toward the medial portions residing in the gray matter, i.e., moving anterogradely down the axon toward the terminals. Research in zebrafish indicates that the process of myelination of axons occurs quickly (Czopka et al., 2013). Oligodendrocytes initiate and complete myelination of a single segment along an axon within just 5 hours (Czopka et al., 2013). The rapid myelination processes could also explain why we did not detect age differences in g-ratios despite the robust increase in *de novo* myelination during this time. Presumably, the short period of time between the initiation and completion of myelin ensheathment of an axonal segment prevented us from capturing axons at a point when the new myelin sheaths were still thin. One benefit of rapid myelination may be the ability of the axon to quickly reach its optimal g-ratio (∼0.77 for axons in the central nervous system; Chomiak and Hu, 2009).

Several studies have demonstrated that variations in either axonal diameter or in myelin ensheathment can generate variability in transmission speed. This variability serves to synchronize the arrival of the signals to the same destination despite differences in fiber length, i.e., isochronicity (Baker and Stryker, 1990; Sugihara et al., 1993; Salami et al., 2003; Lang and Rosenbluth, 2003). We found that conduction velocity was higher in the axons that had to travel further away from CC_FM_ to reach their target cells in Layer V of the Cg1. This suggests a possibility of isochronicity of incoming neural signals to these postsynaptic cells. Moreover, this capability may already be in place by PD15 because the positive relationship between transmission distance and velocity was similar in both age groups. The fact that there was a negligible number of myelinated axons found in the Cg1 of younger animals argues against differential myelination as a mechanism underlying this relationship. Perhaps variation in axonal diameters plays a role, but this remains to be determined.

Developmental improvements in prefrontal function could be due to a number of factors, including the higher axonal transmission speed observed in the current study. There is also documented evidence for increased connectivity between prefrontal and limbic regions (Cunningham et al., 2002), changes in excitatory and inhibitory neurotransmission (Jackson et al., 2001; Floresco and Tse, 2007; Grace et al., 2007), and pruning (Crews et al., 2007), all of which could lead to enhanced neural processing and communication. We noted a trend of an increase in jitter in the older group, which could signify other developmental changes. Higher jitter is often associated with multiple axonal inputs synapsing onto the same cell, especially if these inputs are polysynaptic. However, the stimulation paradigm used for these experiments isolated monosynaptic connections. Therefore, increased variability in neurotransmission time could be due to more monosynaptic connections projecting to the same cell in older animals or to other factors such as calcium availability, neurotransmitter release, opening of the channels, etc. Additional research would be required to distinguish between these different possibilities.

Several limitations of the present study should be considered. First, because we focused on male rats in this initial study, it is unknown whether females show similar developmental changes in prefrontal axons. Second, without a more extensive timeline, we were not able to determine if the increase in myelination of these prefrontal axons between PD15 and PD43 is gradual or sudden. A diffusion tensor imaging study in male rodents shows that FA sharply increases between PD12 and PD18 in the cingulum–an axonal bundle that is adjacent to the corpus callosum (Calabrese and Johnson, 2013). These imaging data suggest that there is a rapid increase in myelination or axonal alignment of cingulum axons. As the CC_FM_ region sampled in our study may contain some of the anterior axons of the cingulum, it will be important in future studies to refine the timeline and determine how rapidly CC_FM_ axons are being myelinated, and if this developmental trajectory differs with sex. Third, as with many developmental studies we must consider the confounding variables of studying these two age groups. Animals under PD21 such as the pre-adolescent animals in our study are usually housed in larger groups with nursing moms, whereas animals older than PD21 such as the adolescent animals in our study have already been separated from their mothers and were housed in pairs or triads. Moreover, the animals herein were shipped from the vendor, and were therefore exposed to this stressor during different developmental time points and with or without nursing moms. Housing conditions and stress hormones are known to impact myelin (Chari et al., 2006; Makinodan et al., 2012). Differential sensitivity to these experiences could have modulated some of the age-related differences in myelin measures, although it is unlikely to fully account for the 5- and 90-fold changes in myelinated axon number observed in the present study. Fourth, our electrophysiological design isolated only part of the axonal pathway projecting to the cells in Layer V. As such, the origin of these axons is unknown and we can only infer that isochronicity was evident before adolescence based on the relationship between transmission distance and speed in younger animals. Future studies using tract tracing techniques could help determine the origin of these axons and would allow for more direct investigation of isochronicity, similar to what has been observed in other brain regions (Baker and Stryker, 1990; Sugihara et al., 1993). Finally, we were not able to measure diameters of unmyelinated axons, as our experimental design only allowed us to visualize myelinated axons. It should be noted that the average and range of diameters of myelinated axons in the CC_FM_ did not change with age despite a substantial increase in the number of myelinated axons, similar to what has been shown in the CC splenium (Kim and Juraska, 1997). It is therefore reasonable to assume that axonal diameters may be similar in unmyelinated and myelinated axons.

In conclusion, the data altogether provide insight into the microstructural changes that occur within prefrontal axons during adolescence and how these developmental changes may affect the speed of electrical signals coming into this brain region. The observed developmental increase in conduction velocity, coupled with a lack of significant change in axonal diameter across development, suggests myelination may be the key contributor to this change in axonal speed. These findings highlight the critical role myelin may be playing in brain maturation just before and during adolescence. Future studies in rodents could allow for direct comparison between the microstructural methods used in the current study with macrostructural imaging techniques such as diffusion tensor imaging (DTI). This would help improve our interpretation of imaging data obtained in humans. This study also provides an important foundation for future studies investigating how toxic substances or environmental disruptions could interrupt normal development and lead to impaired function of these prefrontal circuits.

## Synthesis

Reviewing Editor: Cheryl Sisk, Michigan State University Decisions are customarily a result of the Reviewing Editor and the peer reviewers coming together and discussing their recommendations until a consensus is reached. When revisions are invited, a fact-based synthesis statement explaining their decision and outlining what is needed to prepare a revision will be listed below. The following reviewer(s) agreed to reveal their identity: Janice M Juraska, Douglas Dean.

Reviewers agreed that this is a well-needed study that relates the conduction speed of cortical efferents to their myelination status during the developmental period when cortical myelination is most accelerated - between the juvenile and adolescent period. Specifically, the study examined age-dependent changes of axonal populations extending from the anterior corpus callosum to the medial dorsal prefrontal cortex, in particular cells from the anterior cingulate. The authors find that the conduction velocity is significantly impacted in monosynaptic cells between the anterior corpus callosum and anterior cingulate, with this velocity 1.5-2x as fast in adolescence compared to the pre-adolescence cells. Moreover, transmission distance and latency were not observed to be significantly correlated, however, conduction velocity and distance were positively related, suggesting conduction velocity was higher in axons projecting to a larger distance. G-ratio, average diameter of axons and the range of axon diameters were also not observed to change preadolescence to adolescence. The results suggest that ongoing myelination plays a critical role to neurotransmission speed and that this speed continues to develop into adolescence. Overall, the manuscript is well written, and the findings are of interest.

Reviewers noted many strong aspects to the study; however, there are also some concerns that need to be addressed in a revised manuscript.

1. The potentially most problematic issue is the origin of the animals in the study. In the Methods, it is stated that “Male Wistar rats were ordered from Charles River.” Were animals as young as PD 8-15 shipped (presumably with their mother and litter)? The amount of stress from such a shipment would influence the results, especially the timing since corticosteroids decrease myelination (eg, Chari et al., 2006). Even the adolescent males would be affected by shipping. Ideally, this experiment should have been conducted with animals that were bred and/or born in house. Please be more explicit in the Methods about whether pregnant dams were shipped, whether the animals used in the study were born in the lab, or whether they arrived with their mothers. The shipment of animals is a serious drawback to the study and the effects, especially through maternal behavioral changes, may limit the how well the study will generalize. Therefore, please also include a discussion of the potential confound that the stress associated with shipping could have had on the study, since stress can interfere with myelination and other aspects of morphology as well.

2. The study is framed as myelination being part of the behavioral/functional change that occurs in the prefrontal cortex during adolescence. That may be true, but myelination occurs in other cortical areas as well in the same time frame. There has been a considerable body of work on the splenium of the corpus callosum, which is comprised of axons mainly from the occipital cortex, in several mammalian species. In the rat, Kim and Juraska (1997) found a rapid increase in myelination between PD15 and PD25 and again to PD60 in the splenium as well as stability in axon diameter for myelinated axons between these ages; females were also examined. The cortical axons across areas may be myelinating in a nearly simultaneous manner. In general, the implication throughout the manuscript that the increase in myelination is especially important for behavioral changes seen in prefrontal functioning is overemphasized. The last sentence in the Abstract, in particular, needs to be revised since it is not cognitive speed, but rather cognitive mode, that changes between childhood and adolescence.

3. In describing the conduction velocity results between pre-adolescence and adolescence, it would be informative to include the mean and standard deviation of these velocity measurements within the text (not just Figure 1).

4. Similarly, please include the mean and standard deviations of the cross-sectional areas of the CCFM and number of axons between the pre-adolescence and adolescence ages within the text of the results. Please also include the mean and standard deviations of the g-ratios, average diameter of axons and the range of axon diameters, within the results.

5. In the introduction, the authors described fractional anisotropy as a MRI measure of myelin. One should be careful here, however, as fractional anisotropy is known not to be specific to myelin (see Jones, D. K., Knosche, T. R., & Turner, R. (2013). White matter integrity, fiber count, and other fallacies: the do's and don'ts of diffusion MRI. Neuroimage, 73, 239-254. doi:10.1016/j.neuroimage.2012.06.081). The authors may want to mention this limitation.

6. The authors may also want to include additional developmental studies in their description of studies examining myelination in the context of cognitive development:

a. Chevalier N, Kurth S, Doucette MR, et al. Myelination Is Associated with Processing Speed in Early Childhood: Preliminary Insights. PLoS One. 2015;10(10):e0139897.

b. O'Muircheartaigh J, Dean DC, 3rd, Ginestet CE, et al. White matter development and early cognition in babies and toddlers. Hum Brain Mapp. 2014;35(9):4475-4487.

c. Deoni SC, O'Muircheartaigh J, Elison JT, et al. White matter maturation profiles through early childhood predict general cognitive ability. Brain Struct Funct. 2016;221(2):1189-1203.

7. As the authors describe MRI methods in human subjects, another area of future direction could be comparison of myelin MRI techniques to the measures that are performed here. Comparisons of histological and MRI based measures would help inform on associations observed in humans. The authors may also wish to discuss this point.

8. On a more minor note: the photomicrographs in Figure 2D do not have good resolution; perhaps some arrows with more explanation would help or better photos. Are the coronal section in Figure 2A and the Visual Abstract at the same anterior/posterior level? How was this determined?
